# The Bromides in Inebriety

**Published:** 1882-08

**Authors:** 


### Selections.

### The Bromides in Inebriety.

The withdrawal of alcohol in inebriety is followed by many and often grave nervous disorders, for which the bromides are found most specific. The reflex action of the spinal cord is depressed by this remedy, until the system can accomodate itself to the new conditions. Nervous activity is lowered, and all nerve force is diminished. How far this can be used with safety must be determined in each case. This remedy is literally dangerous in its indiscriminate use for disorders following the withdrawal of alcohol. Its effects are uncertain, and can with difficulty be controlled. They are of degrees merely, ranging all the way from mild sleep, to stupor, unconsciousness, and delirium. Sometimes they act quickly, then their action is delayed and cumulative. Bromide of ammonium is now more commonly used, because it contains more bromine. For the symptons which follow the removal of alcohol, this drug is usually given in forty or fifty grain doses, often repeated, until the patient is quiet. Some physicians give one hundred grains every hour, until sedation is secured. Cannabis indica, hyoscyamus, and digitalis are often combined with this drug, and the effects are still more uncertain. The great danger comes from over sedation, with the consequent hyperaemiaes and anaemiaes, and in cases of inebriety both of these conditions may be present and still further increased by this drug. In some cases bromides may be very dangerous in still further depressing vital energies, that are already worn out. Some years ago I gave the bromides in this state, fearlessly, and without thought of any injury following from them. Occasionally heavy stupor and long continued narcotism would follow, coming on suddenly. Such patients would recover more slowly, and seem more neuraesthenic than others. Other cases after taking one or two hundred grains would become delirious, and cause much trouble, slowly recovering. Some cases would take but little of the bromide, and their restoration would be more positive. Patients themselves will find the bromides of value to check the psychical pain from which they suffer, and take large doses secretly. The experience of many years has taught me to inquire into the history of cases before giving bromides. A case much worn and debilitated, is given small doses of the bromide in large draughts of ginger water. As soon as sedation is apparent this is discontinued. I never give large doses of the bromides for any length of time. Where small doses produce rapid effects I combine it with a tonic, usually cinchonia. Where no effects are apparent, I give warm baths, and very often get the full effect as soon as the bath is over. The remark of Dr. G. M. Beard is confirmed by all experience, viz: "The bromides are powerful remedies, and they may be dangerous as well as powerful, but if we use them wisely, we can obtain and utilize their full power without danger." Certain rules should always govern their administration in inebriety. 1. Never give bromides to stupor, or full bromisation. If stupor follows—use hot baths and general nerve tonics.

2. If the bromides do not act promptly, give small doses at long intervals, and watch for the cumulative effect of all that has been given.

3. Never aim at anything more than limited sedation in these cases with bromide. If it does not follow the administration of three or more drams, try other remedies.

4. Bromides are dangerous remedies in inebriety, not so much from the direct action of the drug, as its influnce on the diseased organism.

